# Comparative and expression analyses of AP2/ERF genes reveal copy number expansion and potential functions of ERF genes in Solanaceae

**DOI:** 10.1186/s12870-022-04017-6

**Published:** 2023-01-23

**Authors:** Jin-Wook Choi, Hyeon Ho Choi, Young-Soo Park, Min-Jeong Jang, Seungill Kim

**Affiliations:** Department of Environmental Horticulture, University of Seoul, Seoul, 02504 Republic of Korea

**Keywords:** AP2/ERF, Transcription factors, Re-annotation, Solanaceae, species-specific duplication, Abiotic stress

## Abstract

**Background:**

The AP2/ERF gene family is a superfamily of transcription factors that are important in the response of plants to abiotic stress and development. However, comprehensive research of the AP2/ERF genes in the Solanaceae family is lacking.

**Results:**

Here, we updated the annotation of AP2/ERF genes in the genomes of eight Solanaceae species, as well as *Arabidopsis thaliana* and *Oryza sativa.* We identified 2,195 AP2/ERF genes, of which 368 (17%) were newly identified. Based on phylogenetic analyses, we observed expansion of the copy number of these genes, especially those belonging to specific Ethylene-Responsive Factor (ERF) subgroups of the Solanaceae. From the results of chromosomal location and synteny analyses, we identified that the AP2/ERF genes of the pepper (*Capsicum annuum*), the tomato (*Solanum lycopersicum*), and the potato (*Solanum tuberosum*) belonging to ERF subgroups form a tandem array and most of them are species-specific without orthologs in other species, which has led to differentiation of AP2/ERF gene repertory among Solanaceae. We suggest that these genes mainly emerged through recent gene duplication after the divergence of these species. Transcriptome analyses showed that the genes have a putative function in the response of the pepper and tomato to abiotic stress, especially those in ERF subgroups.

**Conclusions:**

Our findings will provide comprehensive information on AP2/ERF genes and insights into the structural, evolutionary, and functional understanding of the role of these genes in the Solanaceae.

**Supplementary Information:**

The online version contains supplementary material available at 10.1186/s12870-022-04017-6.

## Background

Plants are exposed to a broad range of biotic and abiotic stresses. They have evolved defense responses to allow them to cope with and adapt to stress [1, 2]. Transcription factors (TFs) are often involved in plant responses to stress and typically bind to specific cis-elements, thereby regulating the expression of the target gene downstream [3, 4]. The APETALA2 (AP2)/ Ethylene-Responsive Factor (ERF) superfamily is one of the largest families of TFs in plants. Members of this family have an essential role in plant responses to diverse stresses as well as in development [5, 6]. The TFs in the AP2/ERF superfamily contain one or more AP2/ERF domains, which have 60–70 conserved amino acids [7]. The family is generally divided into six subfamilies based on domain architectures and specific amino acid residues. These subfamilies are APETELA2 (AP2), AINTEGUMENTA (ANT), ERF, Dehydration-Responsive Element-Binding protein (DREB), ABI3/VP1 (RAV), and Soloist [7, 8]. AP2/ERF genes in the AP2 and ANT subfamilies have two conserved AP2 domains, while members with just one AP2 domain are assigned to ERF or DREB subfamilies. Members with the AP2 domain and the B3 binding domain belong to the RAV subfamily [9], and those with a sequence in the AP2 domain that is distinct from the other subfamilies are in Soloist [7]. For genes in which the domain architecture is identical in different subfamilies, the specific amino acid sequences in the AP2 domain are considered for the classification. Specifically, the TFs that belong to the AP2 subfamily contain 10 amino acid insertions in the AP2 domain region, while those in the ANT subfamily do not have these insertions [10]. The ERF and DREB subfamilies have distinct amino acid residues in the 14^th^ and 19^th^ positions. Proteins in the ERF subfamily have alanine (Ala) and aspartic acid (Asp), and proteins in the DREB subfamily have valine (Val) and glutamic acid (Glu) in the 14^th^ and 19^th^ positions, respectively [8].

The genes in the AP2/ERF superfamily have been studied in several plant species, including *Arabidopsis thaliana, Oryza sativa*, *Zea mays*, *Glycine max,* and *Populus trichocarpa* [7, 11–13]. These genes have also been identified in the Solanaceae family, which includes many economically important crops, notably the tomato (*Solanum lycopersicum*), the potato (*Solanum tuberosum*), and the pepper (*Capsicum annuum*) [14–16]. In these studies, the AP2/ERF genes were first identified and, next, evolution and expression analyses, based on previously published annotations, were performed. However, many important genes were not included in the published annotations and there has been little or no interactive research with updated annotations of AP2/ERF genes in the Solanaceae family [17–21]. For these reasons, we regard comprehensive comparative and functional analyses as indispensable to understanding the AP2/ERF gene families in the Solanaceae.

We have performed annotation updates on Solanaceae AP2/ERF genes and comparative analyses of these AP2/ERF genes with those from *A. thaliana* and *O. sativa*. We identified 2,195 AP2/ERF genes; of these, 368 (17%) were newly annotated. From the results of structural and phylogenetic analyses, we verified that the AP2/ERF genes were grouped into 12 subgroups according to discrete domain architectures; these groups were: A1-A4, B1-B4, AP2, ANT, RAV, and Soloist. Synteny analysis between pepper, tomato, and potato revealed repertory changes of AP2/ERF genes by copy number expansion in specific ERF subgroups of specific species. From our analysis, we showed that these expanded genes in the three Solanaceae species mainly emerged via recent gene duplications in individual species after the completion of divergence of these species. Furthermore, the expression profiles and gene ontology (GO) enrichment test in pepper and tomato revealed diverse putative functions in ERF subgroups under various abiotic stress conditions. We believe that our study can be used to illustrate the evolutionary and expressional characteristics of the AP2/ERF genes in the Solanaceae, and we hope the information will serve as a fundamental genomic resource for further functional and breeding studies on Solanaceae crops.

## Results and discussion

### Re-annotation and characterization of the AP2/ERF gene family in Solanaceae

**Fig. 1:**
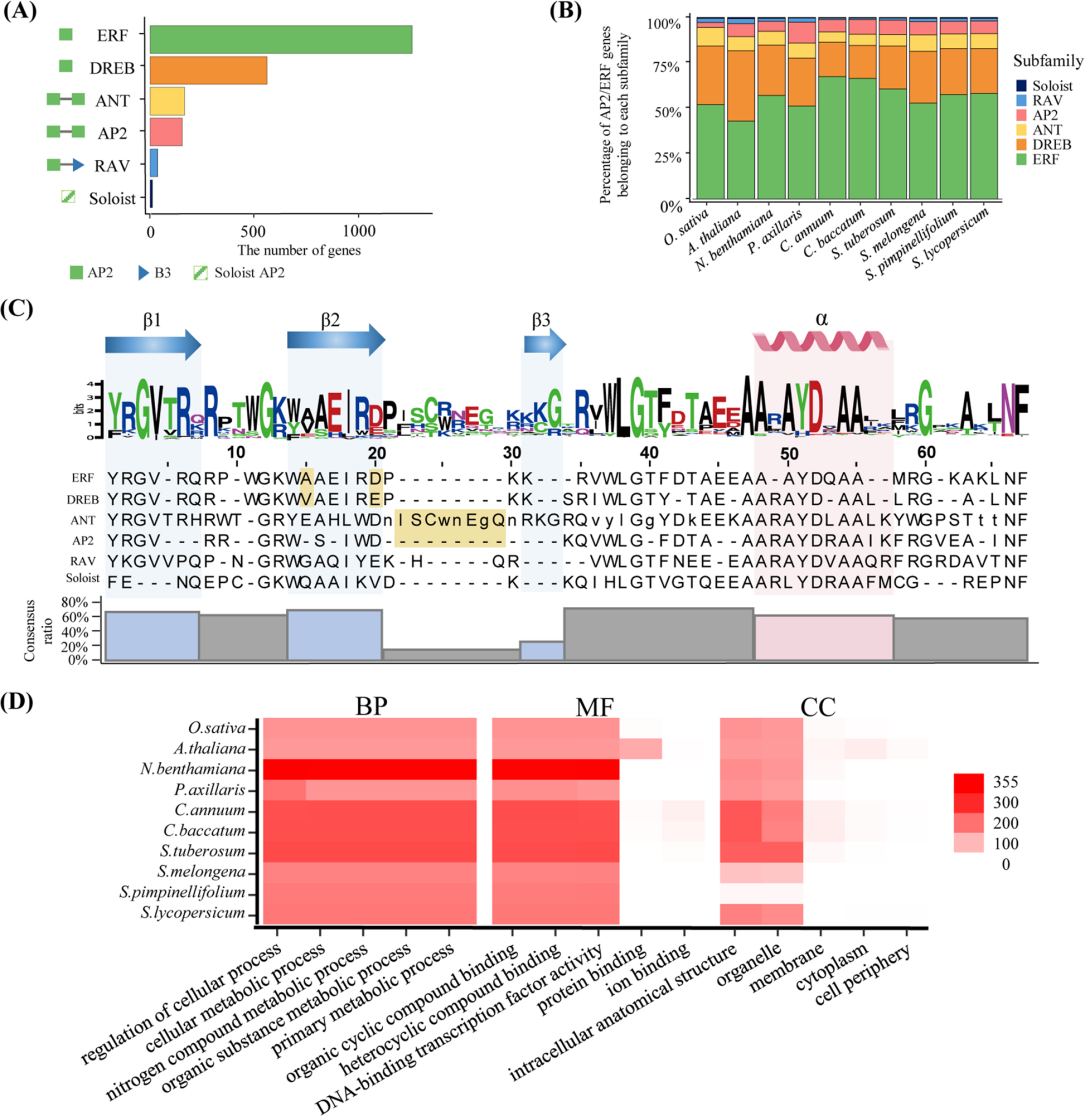
Characteristics of AP2/ERF genes in Solanaceae. **A** The number of AP2/ERF genes from six subfamilies in 10 plant genomes. The domain repertoires of six subfamilies are represented by symbols positioned on the left side of the bar graph. The color of each bar indicates the subfamily. **B** The proportion of genes from subfamilies in the 10 species. The color of each bar indicates the subfamily. **C** Representation of the amino acid sequence and secondary structures in the AP2 domain from 10 species. The x- and y-axes in the diagram of the amino acid logo indicate the position of amino acids in the AP2 domain and the relative frequency of amino acids at each position, respectively. The location of the secondary structure is depicted on the upper side of the amino acid logo. The alignment of amino acid sequences positioned below the logo shows the most conserved amino acids in each position. For the sequence in uppercase, more than half of the genes contain specific amino acids in that position; if this is not the case, the sequence is lowercase. The specific amino acid residues that are critical in distinguishing the subfamily are highlighted in yellow. The bar graph below the alignment shows the consensus ratio of eight divisions considering the secondary structure of the AP2 domain. The colors of the bar display different divisions: pink bar indicates division in α-helix, blue bars indicate divisions in β-sheets, and grey bars indicate the interval regions. **D** Heat map of the number of the top five gene ontology (GO) terms of AP2/ERF genes. Three GO categories are listed over the heat map. The number of GO terms is indicated by the color of the boxes next to the heatmap

We performed a re-annotation of AP2/ERF genes in 10 genomes, specifically eight Solanaceae species, *A. thaliana,* and *O. sativa*. This was done to construct advanced AP2/ERF gene models of these genomes. We identified 2,195 AP2/ERF genes containing 368 (17%) newly annotated genes in these genomes. Of them, 48% of genes (176) were newly annotated based on RNA-seq data or protein evidence, suggesting that these genes are annotated based on high-confident evidence, whereas the other genes could be inactive. The numbers of genes per species ranged from 142 in *A. thaliana* to 355 in *Nicotiana benthamiana* (Table 1). *A. thaliana* had the fewest newly annotated genes (1) and *Capsicum baccatum* had the most (74). We investigated the structures of the updated AP2/ERF genes to characterize the general features of AP2/ERF genes in Solanaceae. Following the rule of dividing subfamilies described previously [7], we divided the AP2/ERF genes into six subfamilies according to the number of AP2/ERF domains and bases coding for specific amino acid residues: ERF, DREB, ANT, AP2, RAV, and Soloist (Fig. 1A). Specifically, 1,263 genes were classified into the ERF subfamily, which comprises one AP2/ERF domain and codes for Alanine and Aspartate in the 15^th^ and 20^th^ positions, respectively. The proportion of genes in the subfamilies indicates that most were in the ERF subfamily in the Solanaceae (Fig. 1B). As in *Vitis vinifera* and *G. max,* in which the ERF subfamily is a dominant group, more than half of AP2/ERF genes in the Solanaceae family were classified into the ERF subfamily [13, 22].

**Table 1:** Numbers of re-annotated AP2/ERF genes in the eight Solanaceae plants studied here, and in *Arabidopsis thaliana* and *Oryza sativa*

Species	Previously annotated genes	Newly annotated genes	Total
*Oryza sativa*	163	14	177
*Arabidopsis thaliana*	141	1	142
*Nicotiana benthamiana*	323	32	355
*Petunia axillaris*	187	32	219
*Capsicum annuum*	181	67	248
*Capsicum baccatum*	173	74	247
*Solanum tuberosum*	212	42	254
*Solanum melongena*	153	23	176
*Solanum pimpinellifolium*	127	59	186
*Solanum lycopersicum*	167	24	191
Total	1827	368	2195

We also explored structural diversity within subfamilies and examined the coding sequence for specific amino acids in the AP2/ERF domain. As discussed, subfamilies of AP2/ERF genes code for specific amino acid residues in specific positions, and the proteins encoded for by the AP2/ERF domain in the Solanaceae family also had amino acid differences according to subfamily in positions 15, 20, and 22–29 (Fig. 1C). Specifically, the Soloist subfamily included distinct amino acid sequences of the AP2/ERF domain compared to the other subfamilies [23]. We also investigated the secondary structure encoded by the AP2/ERF domain related to the essential role of binding to the GCC box. Consistent with other findings, the AP2/ERF domain coded for three β-sheets and one α-helix: positions 1–7, β1; 14–20, β2; 31–33, β3; 48–57, α (Fig. 1C) [24]. Based on the region of the four secondary structures (three β-sheets and one α-helix), we divided the AP2/ERF domain into eight divisions and calculated the consensus score of each division to compare the conservation degree among subfamilies. We found overall conservation of most of the regions, including the four secondary structures, with the exception that we observed low conservation of the fourth division (14%) among subfamilies. These results suggest that the structural diversity between subfamilies occurred due to the divergence in specific residues and divisions, which may contribute to the functional diversity of AP2/ERF genes in Solanaceae.

Functional annotation through GO analysis was performed to shed light on the putative functions of the AP2/ERF genes in the Solanaceae family. The 2,166 (99%) GO terms for AP2/ERF genes were classified into three categories based on biological processes, molecular functions, and cellular components (Fig. 1D). The predominant GO descriptions in three categories were “regulation of cellular process” (99%; 2,139 genes), “organic cyclic compound binding” (97%; 2,093 genes), and “intracellular anatomical structure” (73%; 1,576 genes). These results were consistent with previous studies, in which it was concluded that functions of AP2/ERF genes were related to regulating target genes [25]. Taken together, our analyses display the overall characteristics of updated AP2/ERF genes in the Solanaceae family by investigating repertoires of the subfamily, amino acid composition encoded within the AP2/ERF domain, and functional prediction of AP2/ERF genes.

### Phylogenetic relationship and copy number expansion of ERF genes in Solanaceae

**Fig. 2:**
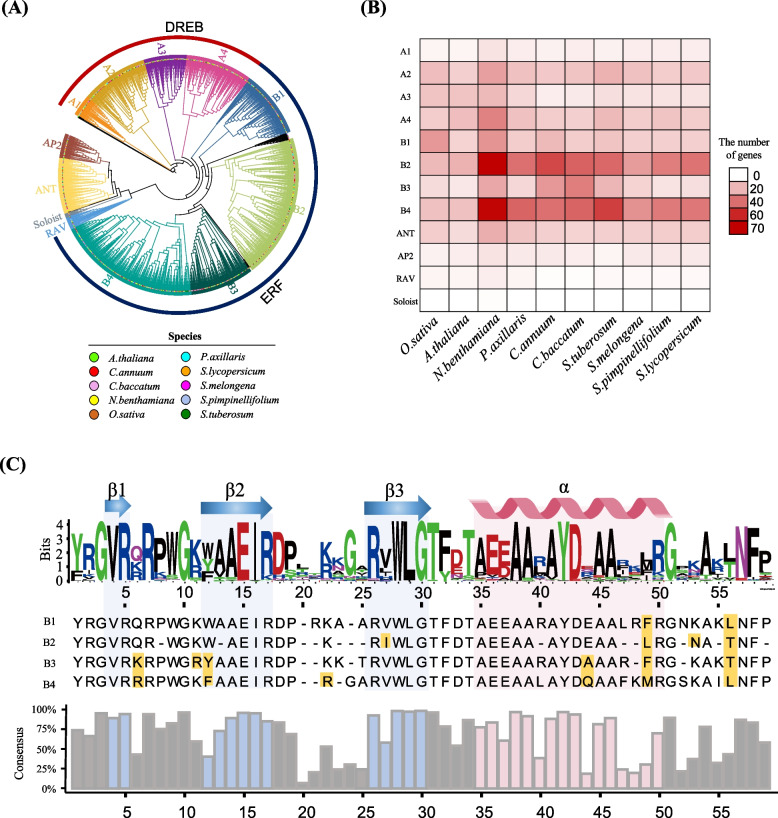
Phylogenetic relationship and subgroup classification of AP2/ERF genes in 10 species. **A** Phylogenetic tree, in which species are represented by different colors of dots on the end of the branch. The color of each branch and the strap outside of colored dots display different subgroups. The outer ring identifies ERF subgroups (B1-B4) and DREB subgroups (A1-A4). **B** Heat map showing the number of AP2/ERF genes in subgroups from each species. **C** The conserved amino acid sequence of AP2 domain in ERF subgroups. The stacked logo depicts multiple amino acids in a specific position. The height of the logo indicates the frequency of amino acids. The letter under the logo indicates the most conserved amino acid in each position. The different conserved residues between ERF subgroups are highlighted in yellow. The position of α-helices and β-sheets are displayed on the upper side of the logos. The calculated consensus ratio of each position is shown as the bar with the color of the secondary structure of the AP2 domain

We constructed a phylogenetic tree with the updated AP2/ERF genes of the 10 species to explore the evolutionary relationships of AP2/ERF genes in Solanaceae. We divided AP2/ERF genes into 12 subgroups, based on their phylogeny and the previously described rule for dividing subfamilies (Fig. 2A) [7–10]. The updated AP2/ERF genes within the same subfamily were distinctly clustered into subgroups (Fig. 2A). For example, genes from the ERF and DREB subfamilies were clearly clustered into B (B1-B4) and A (A1-A4) subgroups, respectively. We then compared copy numbers of AP2/ERF genes among subgroups from each species (Fig. 2B). Overall, the copy numbers of genes in subgroups among species were similar. However, genes in subgroups B2 and B4 were specifically abundant in Solanaceae species. This suggests that genes belonging to these subgroups from the ERF subfamily in Solanaceae were expanded in a lineage-specific manner.

Different amino acid residues coded for by the AP2/ERF genes within the ERF subfamily have apparently led to divergent DNA-binding specificities [26]. We extended these findings and investigated the amino acid residues of the AP2/ERF domain of genes in B1-4 subgroups to explore structural diversity within the ERF subfamily. The B1-4 subgroups had significant differences in specific positions of amino acids in the protein. Position 49 had phenylalanine, leucine, phenylalanine, and methionine, in B1-4 subgroups, respectively, and position 56 had leucine, threonine, threonine, and leucine, respectively. These results concur with those of Shoji et al. [26], and indicate that these specific amino acids within ERF subgroups B1-4 contributed to structural diversification, resulting in the construction of distinct lineages of ERF genes [7, 26]. Taken together, our findings provide an overview of the phylogenetic relationship and lineage-specific structural features of AP2/ERF genes that will serve as a fundamental genomic resource for future genetic and functional research.

### Copy number expansion of ERF genes through recent gene duplication among Solanaceae

We next verified the physical position of the AP2/ERF genes in the pepper, tomato, and potato. Most of the AP2/ERF genes in these species were evenly distributed throughout the ends of chromosomes with a few exceptions (Fig. S1). These exceptions were: the genes belonging to the ERF subgroups, abundant in Solanaceae, formed a tandem array on specific chromosomes; genes in the B2 subgroup were clustered on the short arm of chromosome 1 in all three species; the genes in the B3 subgroup formed a tandem array on the short arm of chromosome 4 in pepper.

**Fig. 3:**
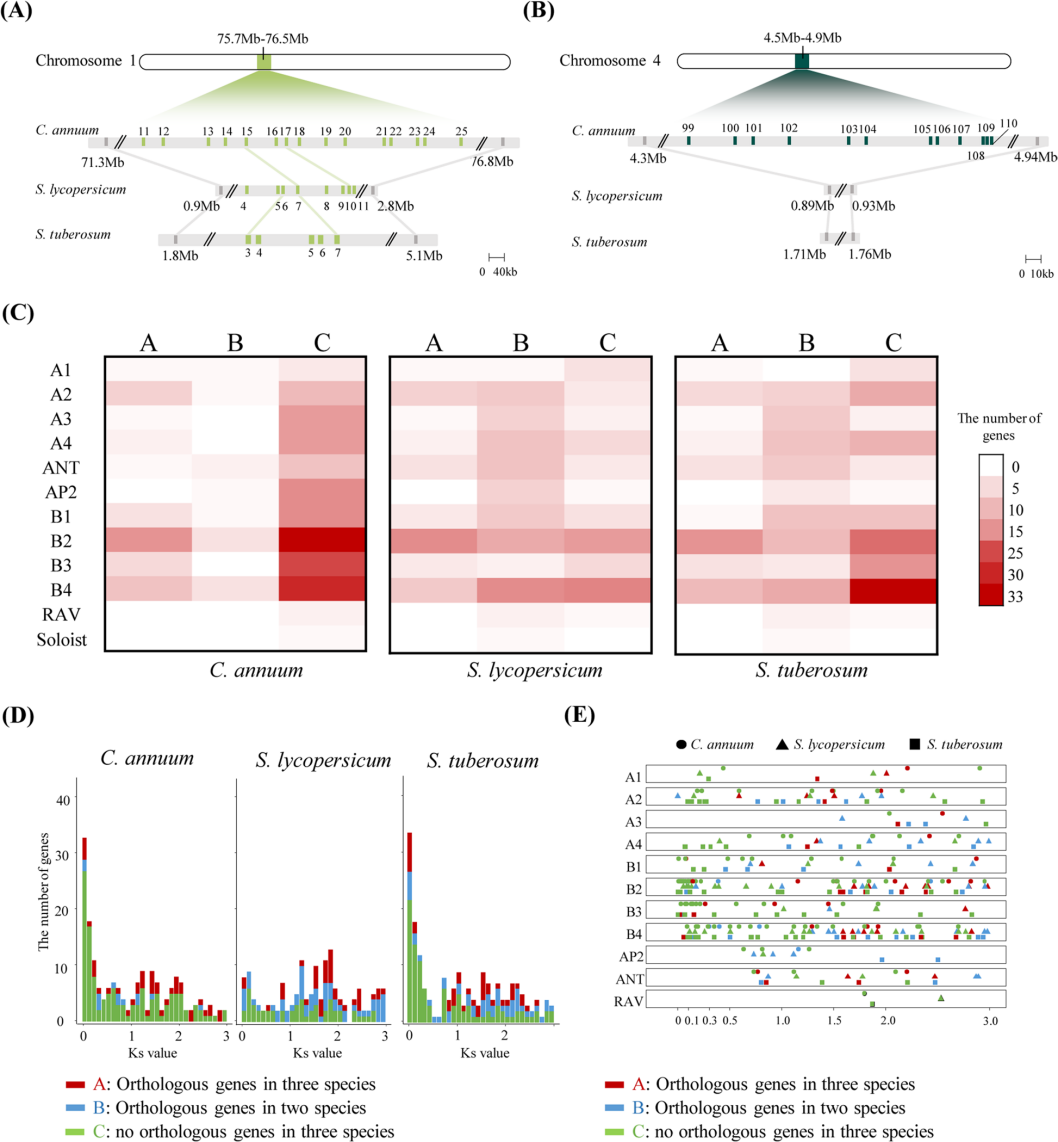
Comparison of the syntenic region and duplication history of the AP2/ERF pepper, tomato, and potato genes. **A**-**B** Micro-synteny analysis of AP2/ERF genes in the subgroup B2 **A** and B3 **B** on chromosomes 1 and 4. The gene IDs in the syntenic region are represented and the orthologous gene is linked with the line. The genes colored with grey indicate orthologous genes that are not AP2/ERF genes. **C** Heat map of the number of genes with orthologous relationships. The number of genes having orthologs in three, and two species are displayed on A and B, respectively. The number of genes with no orthologs is represented on the **C**. **D** Histogram showing the duplication history of AP2/ERF genes. The color of the bar indicates an ortholog relationship between the three species. **E** The distribution of duplication history of AP2/ERF. The shapes and colors indicate species and ortholog relationships between the three species, respectively

We further explored possible mechanisms for the evolution of the AP2/ERF genes in Solanaceae by examining the micro-syntenic region for the subgroups of these species (Fig. 3A and 3B). The copy numbers of genes belonging to the B2 and B3 subgroups were quite variable in the syntenic region of the three genomes, mainly due to the abundantly clustered genes in the B2 and B3 subgroups of pepper. Most of the genes were not in orthologous relationships within the three genomes. These suggest that the AP2/ERF genes in pepper that are in the B2 and B3 subgroups expanded in chromosomes 1 and 4.

When we performed synteny analyses for all AP2/ERF genes, 70%, 33%, and 50% of AP2/ERF genes were not in an orthologous relationship with genes of the pepper, tomato, and potato, respectively. This indicates that species-specific genes in the Solanaceae may have generated differences in the AP2/ERF gene repertories (Fig. S2 and Fig. 3C) [27]. Of these species-specific genes, 69% of the pepper genes, 68% of the tomato genes, and 70% of the potato genes were in ERF subgroups. In particular, 33 (65%) of the pepper genes in subgroup B2 and 24 (83%) of the genes in subgroup B3 had no ortholog in other species. These imply that many genes in specific ERF subgroups may have undergone species-specific gene duplication in specific chromosomes.

To verify whether the AP2/ERF genes without orthologous genes mainly emerged after the divergence of Solanaceae species, we estimated the duplication period of AP2/ERF genes in the pepper, tomato, and potato genomes through calculation of the synonymous substitution rates between duplicated AP2/ERF gene pairs (Fig. 3D). We observed 62 AP2/ERF genes in the pepper with Ks values < 0.3; 84% of these genes had no orthologous relationship with either the tomato or potato. Likewise, eight genes in the tomato and 34 genes in the potato had Ks values < 0.1, and 25% and 65% of these genes, respectively, had no orthologous relationship with the other two species. Given the divergence time between the pepper and *Solanum* species (0.3 Ks) and between the tomato and potato (0.1 Ks) [28], it may be that recent species-specific duplication of AP2/ERF genes occurred mainly in the pepper and potato. We investigated the Ks distribution of AP2/ERF genes for each subgroup in the three species (Fig. 3E). We observed that 58 (93%) of pepper AP2/ERF genes with Ks values < 0.3 belonged to ERF subgroups (B1-4). Most of these genes (84%) did not have an orthologous relationship with the other two species. In tomato and potato, respectively, 75% and 82% of AP2/ERF genes with Ks values ​​between 0 and 0.1 belonged to ERF subgroups. Most of these genes in the potato (64%) did not have an orthologous relationship with the other two species. These suggest that the repertory of AP2/ERF genes among the three genomes differentiated mainly due to the emergence of species-specific AP2/ERF genes, especially those in ERF subgroups. This occurred through recent gene duplications after the divergence of the three Solanaceae species.

### Expression and functional investigation of AP2/ERF genes in pepper

AP2/ERF genes regulate plant response to abiotic stress [29]. We conducted expression analyses for all pepper genes, including the newly identified AP2/ERF genes, to learn more about their roles in abiotic stress. The expression profiles of pepper AP2/ERF genes were acquired from RNA-seq data in a time course (data were taken at 3, 6, 12, 24, and 72 h after the imposition of stress) to four stresses (cold, heat, mannitol, or salt). By comparing the data from plants under stress with the unstressed control, we identified the following numbers of differentially expressed genes (DEGs): 10,720 under cold stress, 9,992 under heat stress, 3,542 under mannitol stress, and 3,194 under salt stress. Of these, 70, 48, 31, and 46 were AP2/ERF genes that were differentially expressed during cold, heat, mannitol, and salt stress, respectively (Fig. S3 and Table S3). Most of the AP2/ERF DEGs (56%) were in the ERF subgroups: 33 were in B1, 30 were in B2, 14 were in B3, and 34 were in B4. This result suggests that many AP2/ERF genes in ERF subgroups B1-4 participate in the stress response (Fig. S3) [14, 30–32]. In agreement with our suggestion, it has been reported that *CaPF1* (caAP2_140) was up-regulated under cold stress [33].

**Fig. 4:**
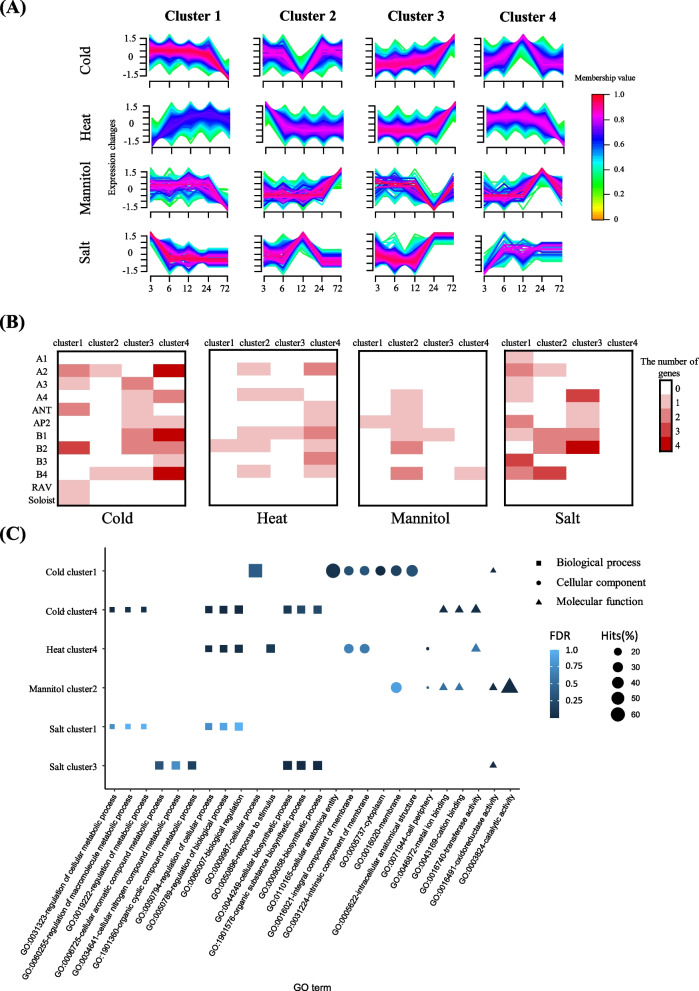
Transcriptome analyses with gene ontology (GO) enrichment test in differentially expressed genes (DEGs) of pepper under abiotic stress. **A** For each stress, expression patterns of whole DEGs, including AP2/ERFs are represented in four clusters. **B** Heat map showing the distribution of AP2/ERF genes in each subgroup. **C** Dot plot showing the top five GO descriptions abundance in specific clusters. The shape and size of the symbols illustrate the type and frequency of the GO description, respectively

We then performed an expression clustering analysis to investigate expression patterns of pepper DEGs including both AP2/ERF and others under abiotic stress conditions. The DEGs were divided into four different clusters (C1-C4) for each stress condition (Fig. 4A). We detected abundant AP2/ERF DEGs in C1 and C4 under cold stress, in C4 under heat stress, in C2 under mannitol stress, and in C1 and C3 under salt stress (Fig. 4B). This represents that the AP2/ERF genes and other pepper genes in these clusters may interact with each other for specific functions under these conditions.

We next conducted GO-enrichment analysis for the genes in the six of 16 clusters mentioned above, which contained abundant AP2/ERF DEGs (Fig. 4C). This was done to gain some insight into the functions of AP2/ERF genes in pepper. We surveyed enriched GO terms in the clusters. We detected many GO terms in the clusters related to regulation, and it may be that these genes have regulatory roles during abiotic stress. For example, GO terms such as “regulation of cellular process (GO:0050794)”, “regulation of biological process (GO:0050789)”, and “biological regulation (GO:0065007)” were enriched in cold cluster 4 and in salt cluster 1, and many of them were included in the ERF subgroups (B1-4). This is consistent with previous studies that AP2/ERF genes in the ERF subgroup are important in the regulation of plant resistance to abiotic stress [30, 33, 34]. Specifically, it is known that *CaPF1* of pepper in the ERF subgroup containing “biological regulation” (GO:0009873) promoted cold tolerance by binding to both sequences of GCC and DRE/CRT (dehydration-responsive element/C-repeat) in PR and COR genes [33]. At the time this work was done because only a few functional AP2/ERF genes had been reported, the pepper genes that had an orthologous relationship with functional tomato genes were analyzed. The purpose was to better understand the function of genes in the ERF subgroups in pepper under salt stress. The CA.PGAv.1.6.scaffold790.71, an orthologous tomato gene of *TSRF1*, known for improving salt resistance, had the child GO description of biological regulation (GO:0009873) [35]. Thus, it seems likely that the pepper AP2/ERF genes in cold cluster 4 and salt cluster 1 are involved in regulatory mechanisms associated with abiotic stress. Taken together, our analyses provide overall expressional and functional features of pepper AP2/ERF genes, that will build a broad understanding of these genes and enhance future studies.

### Transcriptome analyses of AP2/ERF tomato genes

**Fig. 5:**
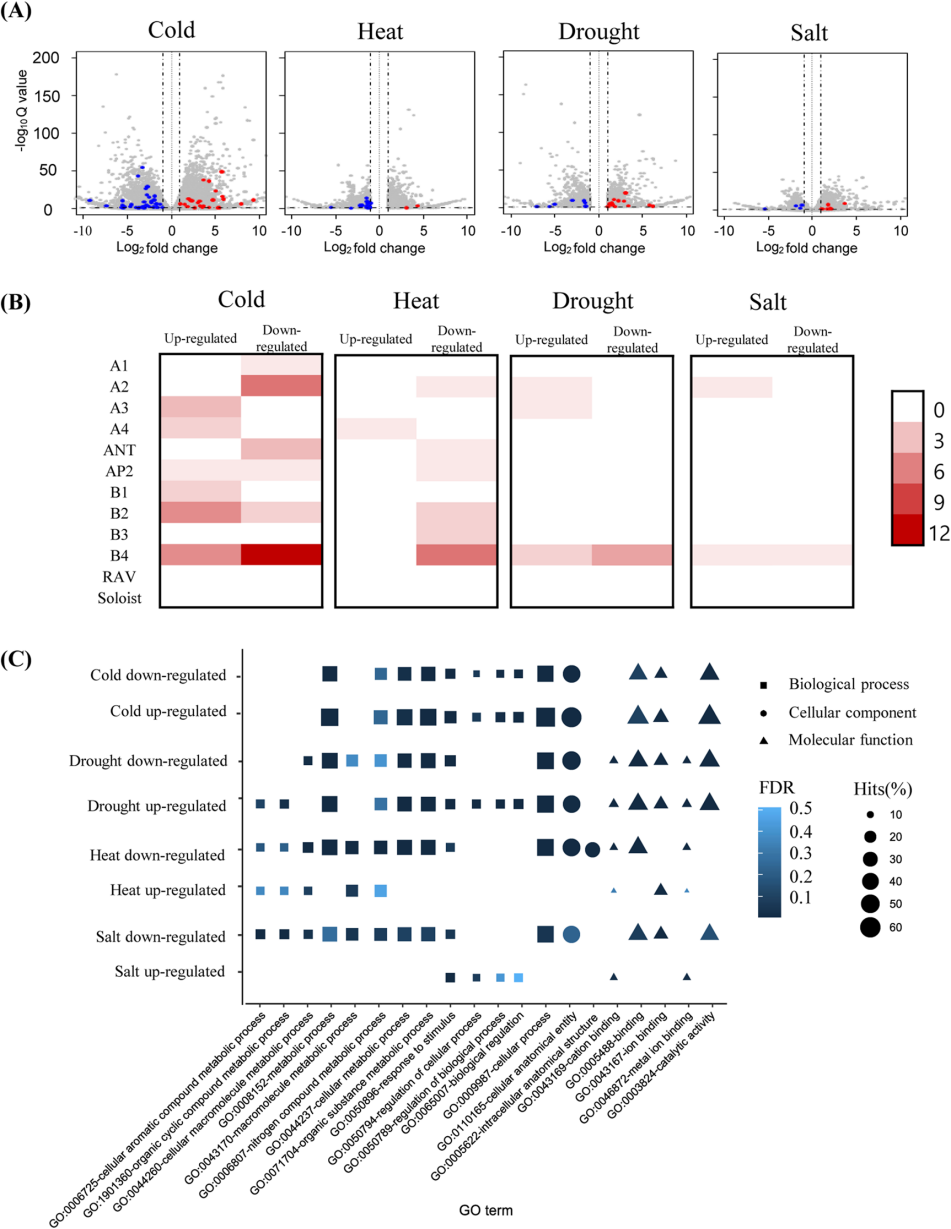
Expression and putative functions of AP2/ERF genes in tomato exposed to abiotic stress. **A** Differentially expressed genes under four abiotic stresses are shown as a volcano plot. The color of scattered points represents the expression difference of AP2/ERF genes (grey: whole genes; red: up-regulated AP2/ERF DEGs; blue: down-regulated AP2/ERF DEGs). **B** Heatmap showing the number of AP2/ERF DEGs in each subgroup. The scale bar on the right side of the heatmap indicates the heatmap value. **C** Depiction of results of the GO-enrichment test. The top five enriched GO terms in each group are listed under the plot. The different shapes and sizes of symbols indicate different categories and the frequency of GO descriptions (DEGs; differentially expressed genes; GO, gene ontology)

We next examined the expression profile of tomato genes under cold, drought, heat, and salt stress. We examined the whole genes, including the newly-annotated AP2/ERF genes, using the RNA-seq data. The following DEGs were identified in different stress conditions: 8,975 under cold stress, 2,698 under drought stress, 4,643 under heat stress, and 2,651 under salt stress. Of these DEGs, 60, 19, 23, and 11 AP2/ERF were identified in tomato under cold, drought, heat, and salt stress, respectively (Fig. 5A and Fig. S4). We detected that many AP2/ERF DEGs were included in ERF subgroups as pepper DEGs (B1:4, B2:16, B3:6, and B4:37). To compare the expression level of AP2/ERF with other genes in the tomato under abiotic stress, we classified DEGs into two groups: up-regulated and down-regulated (Fig. 5B). We verified up-regulation of the expression of *SlDREB2* (slAP2_161) under salt stress, as has been reported previously [36]. This also validated our analyses of the expression of tomato AP2/ERF genes (Fig. S4).

We performed a GO-enrichment test for all genes including AP2/ERF DEGs in the eight groups (Up- and down-regulated groups in four abiotic stresses) (Fig. 5C). Diverse functional descriptions appeared in specific groups. For example, metabolism-related GO terms such as “metabolic process (GO:0008152)”, “cellular metabolic process (GO:0044237)”, and “nitrogen compound metabolic process (GO:0006087)” were present in six groups (down- and up-regulated under cold stress, down- and up-regulated under drought, down-regulated under heat stress, down-regulated under salt stress). Binding-related GO terms such as “binding” (GO:0005488), “ion binding” (GO:0043167), “metal ion binding” (GO:0046872), and “cation binding” (GO:0043169) were enriched in six groups (down-and up-regulated genes under cold stress, down- and up-regulated genes under drought, down-regulated genes under heat stress, down-regulated genes under salt stress). This suggests that tomato genes, including AP2/ERF genes, are involved in diverse functions under abiotic stresses. The response-related GO term “response to stimulus” (GO:0050896) was enriched in seven groups (down-and up-regulated genes under cold stress, down- and up-regulated genes under drought, down-regulated genes under heat stress, down- and up-regulated genes under salt stress). This result is consistent with previous studies that AP2/ERF genes are involved in stress response mechanisms. Specifically, AP2/ERF genes in ERF subgroups (B1-4) are key regulators for several stresses, such as jasmonate (JA), ethylene, salt, drought, and cold treatments [37]. *JERF3* in the tomato, which belongs to ERF subgroups, had the child term of response to stimulus (GO:0009873) and binds to the ethylene/JA responsive GCC box, thereby enhancing salt tolerance [37]. These results suggest that tomato AP2/ERF genes belong to ERF subgroups (B1-4) and associate with stress response genes.

## Conclusions

In the past, annotations have been broadly performed. However, researchers have highlighted the need for analyses of gene families with updated annotation and suggested that such analyses will provide new insight into the evolutionary and functional characteristics of genes [18–21]. In this study, we performed a re-annotation of AP2/ERF genes in eight Solanaceae genomes as well as the genomes of *A. thaliana* and *O. sativa*, and enable to conduct comparative, evolutionary, and functional analyses of the AP2/ERF genes, providing insights into comprehensive structural and functional characteristics of Solanaceae AP2/ERF genes. In particular, our data reveals that there has been species-specific copy number expansion of AP2/ERF genes in ERF subgroups B2-4. We found that genes in ERF subgroups form a tandem array in pepper, tomato, and potato chromosomes. Most of these genes do not have orthologs among the three species: most of the genes without orthologs in ERF subgroups were specific to their species and emerged through recent gene duplication after the divergence of the species. This could explain the different AP2/ERF gene repertories in the Solanaceae family. Based on transcriptome analyses and investigation of functional genes and their orthologs, our findings suggest that genes in ERF subgroups are involved in response mechanisms and interact in the response to abiotic stress. Furthermore, we investigated the similarity of expression patterns between orthologous genes in pepper and tomato (Fig. S5). We found that the expression of orthologous genes had a low correlation under cold, heat, and salt stresses, probably due to different experimental conditions in RNA-seq data, such as differences in the developmental level of plant materials or growth conditions. Consequently, our study provides a comprehensive understanding of structural characteristics, evolutionary history, and potential functions of AP2/ERF genes in the Solanaceae family.

## Materials and methods

### Identification and re-annotation of AP2/ERF genes in 10 species

We downloaded publicly available genomic resources and RNA-seq data of *A. thaliana* [38], *O. sativa* [39], and eight other Solanaceae crops: *N. benthamiana* [40], *Petunia axillaris* [41], *C. annuum* [42], *C. baccatum* [28], *S. tuberosum* [43], *S. melongena* [44], *S. pimpinellifolium* [45], and *S. lycopersicum* [46] (Table S1). For re-annotation of AP2 genes, we operated TGFam-Finder v1.20 [47] with “260,000” and “110,000” for “EXTENSION_LENGTH” and “MAX_INTRON_‌LENGTH” considering maximum gene and intron lengths of AP2/ERF genes in published annotations, respectively. The tab-separated value (TSV) files of AP2/ERF genes were generated from InterproScan 5 (-f tsv, -appl Pfam) [48], which were used for “TSV_FOR_DOMAIN_‌IDENTIFICATION”. The AP2 domain, which was indicated as “PF00847” from the Pfam database was utilized for “TARGET_DOMAIN_ID”.

All re-annotated genes were given new names instead of using locus tag names. In this, we considered species names and the physical position in chromosomes such as “atAP2_1” (Table S2). In cases of the AP2/ERF genes in *A. thaliana*, *O. sativa*, *S. lycopersicum*, and *S. tuberosum*, we matched previously assigned gene names to new names.

### Structure of AP2/ERF genes

The tab-separated value (TSV) files generated by InterproScan 5 (-f tsv, -appl Pfam) [48] according to the Pfam database were used to identify the domain structures of the updated AP2/ERF genes. The domain region of AP2/ERF genes was aligned by the MAFFT program (with default parameter) [49]. We trimmed inconclusive sequences using trimAL v1.4 (gt: 0.05) [50]. To obtain more accurate information for further analyses, we excluded domains with high e-values (> 1e-5) or that overlapped with the AP2/ERF domain.

### Amino acid sequence composition within AP2/ERF domain

We wished to represent graphically the amino acid composition of all AP2/ERF domains in 10 species with a multiple sequence alignment. For this, we utilized a WebLogo program [51]. We used EMBOSS Cons programs (Plurality 0.1, default option) [52] to estimate the consensus sequence of the AP2/ERF domain according to subfamily. The default option, Jpred, [53] predicted the secondary protein structure of the AP2/ERF domain by multiple alignments (JNETPSSM model). From the protein structure, we divided the AP2/ERF domain into eight divisions. We calculated the average consensus scores of each division by multiple alignments of the complete sequences information management system (MACSIMS) in Jalview programs [54].

### Gene Ontology (GO) analysis

We used the OmicsBox tool (v 1.4) [55] to conduct a functional annotation of APE/ERF genes in Solanaceae. With an e-value cut off of 1e-3 as the default setting in the OmicsBox tool, BLASTP [56] was used to match APE/ERF protein sequences to the National Center for Biological Information (NCBI) non-redundant proteins database (nr v5). We combined the result of InterProScan [48] with BLAST results for prediction. We used the default parameters of Blast2GO Mapping and Blast2GO Annotation [57] to match GO terms and subdivided the results of GO analysis into three categories: biological process, molecular function, and cellular component. We visualized the top five GO terms in level 3 of each category with a heatmap.

### Classification and phylogenetic analysis of AP2/ERF genes

The 1937 AP2/ERF genes with intact AP2 domain(s) in 10 species were aligned by MAFFT v7.470 [49] and the alignments were trimmed by trimAL v1.4 (-gt: 0.5) [50]. We constructed a maximum likelihood tree with 500 rapid bootstraps in random parsimony (-m PROTGAMMAJTT -p 12345-× 12345 -#500) via RAxML v8.2.12 [58], which predicted the PROTGAMMAJTT model as the best model (-m PROTGAMMAAUTO -p 12345). The constructed mid-point rooted tree was represented by the Interactive Tree of Life (iToL v5). The AP2/ERF genes were clustered into 12 subgroups. These subgroups were distinguished by the domain architecture and specific residues (A1-A4, B1-B4, AP2, ANT, RAV, and Soloist).

### Chromosomal location and synteny of the AP2/ERF genes

The chromosome distribution of newly annotated genes was obtained from GFF files generated by TGFam-Finder v1.20 [47]. The MapChart [59] was used to visualize the diagram illustrating the chromosomal location of AP2/ERF genes except for unanchored scaffolds. We represented the subgroups of all genes in the phylogenetic tree with different colors.

Syntenic analyses were conducted on pepper (*C. annuum*), tomato (*S. lycopersicum*), and potato (*S. tuberosum*) AP2/ERF genes. We used BLASTP [56] for an all-by-all comparison to detect putative orthologous gene pairs. To find orthologous syntenic chains including gene location information that was generated by TGFam-Finder v1.20 [47], we utilized MCScanX programs [60]. The genomic positions of each gene pair were visualized with RIdeogram packages in R software [61].

### Duplication and synonymous substitution rates (Ks) value analysis

We utilized the DupGen_Finder pipeline [62] to identify duplicated AP2/ERF gene pairs as described in previous studies. The coding sequences of gene pairs were probabilistic multiple aligned with PRANK (-codon) [63]. We calculated the gene pairs of the synonymous substitution rates (Ks) with the KaKs_calculator v2.0 (-m MYN) [64]. We visualized the gene pairs (Ks value < 3) with ggplot2 [65] in the R package.

### Expression analyses of pepper and tomato AP2/ERF genes

We acquired RNA sequencing (RNA-seq) data from the leaves of pepper [66] and tomato (SRR7652567, SRR7652566, SRR7652565, SRR7652564, SRR7652571, SRR7652570, SRR7652569, SRR7652568, SRR7652563, SRR15410554, SRR15410555, SRR15410556, SRR15410551, SRR15410552, SRR15410553, SRR15607561, SRR15607560, SRR15607558, SRR15607557, SRR15607556, and SRR15607555) under diverse abiotic conditions to evaluate the expression patterns of the AP2/ERF genes in these species. The RNA-seq data from the pepper was acquired under cold, heat, salt, and mannitol stress at various times (3, 6, 12, 24, and 72 h) after the imposition of stress. Tomato RNA-seq data was collected from cold, heat, drought, and salt treatments without specific time points. Three biological replicates were performed.

The raw FASTQ files by CLC Assembly Cell (CLC Bio, Aarhus, Denmark) were trimmed to remove low-quality RNA-seq results. The files generated were mapped to each reference genome of *C. annuum* and *S. lycopersicum* by HISAT2 (-dta -x) [67]. The fragment per kilobase of transcript per million mapped reads (FPKM) values of whole genes was calculated by the StringTie (-e, -B -G) [68], including the newly annotated pepper and tomato AP2/ERF. Python scripts (prepDE.py) were used to convert FPKM values to read counts. We used DESeq2 in R software [69] to identify DEGs with the following criteria: log_2_ FoldChange > 1 or < -1, and adjusted *p*-value < 0.05.

Clustering analysis was performed on all pepper genes by Mfuzz in the R package [70] to inspect AP2/ERF expression arrangement. Based on the k-means algorithm, four clusters with up-regulated genes and down-regulated genes were identified. We also arranged all DEGs in the tomato into two groups: up- or down-regulated groups, in each stress treatment. We performed GO annotation and Fisher’s exact test (false discovery rate [FDR] p-value ≤ 0.01) for each cluster or group with OmicsBox v1.4 [55] to test GO-enrichment.

As we considered the number of functional genes in the pepper to be deficient, we surveyed orthologs of functional genes. We used Exonerate v2.2.0 (-model protein2genome, -showtargetgff yes, -showquerygff yes, -querytype protein, -targettype DNA) to detect the candidate regions of the orthologs [71].

### Investigation of similarity of expression pattern

We examined FPKM values of orthologous genes between pepper and tomato under cold, heat, and salt stress (Fig. S5). As we can not check the time points of treatment in tomato RNA-seq data, we calculated the average FPKM values between five time series of pepper FPKM values. We used pearson method to test correlation between pepper and tomato expression patterns.

## Supplementary Information

**Additional file 1: Table S1.** Genomic resources of 10 plant species.** Table S2.** Data for re-annotated AP2/ERF genes.** Table S3.** log_2_ (Fold change) values of Capsicum annuum in stress condition.** Table S4.** log_2_ (Fold change) values of Solanum lycopersicum in stress condition.

**Additional file 2: Fig. S1. **Chromosome allocation of pepper, tomato and potato AP2/ERF genes. White rectangular boxes indicate chromosomes.** Fig. S2. **Syntenic analyses of all pepper, tomato and potato chromosomes. Colored rectangles represent the chromosomes (chr) of each species. The genes with orthologous relationships among the three species are linked with lines. The line color indicates details of the orthologous relationships, as indicated.** Fig. S3. **Expression profiles of pepper AP2/ERF genes under various abiotic stresses. **A** Heat map showing the relative differences of expression of AP2/ERF genes under abiotic stress. The heat map values (log_2_ fold-change) are calculated by FPKM (abiotic stress) /FPKM (control). The names of the AP2/ERF genes are displayed next to the heat map. The normalized values of the heat map are represented in the scale bars, positioned on the right side of the heat map; green implies a low level of expression, whereas red represents a high level of expression. **B** The distribution of AP2/ERF DEGs in the subgroups is illustrated in the heat map (inset). FPKM, fragments per kilo base of exon per million mapped fragments.** Fig. S4. **The expression value of tomato AP2/ERF genes under various abiotic stresses. **A** The heat map represents the value of expression of AP2/ERF genes under abiotic stress. The values are calculated by log_2_ fold-change of tomato AP2/ERF genes and fold-change values are calculated by dividing FPKM from each stress to control (C: Cold, D: Drought, H: Heat, S: Salt). The colored bar positioned on the right represents the following range of expression levels:-3 (green) to +3 (red). **B** The number of tomato AP2/ERF DEGs in are displayed in heat map (inset). FPKM, fragments per kilo base of exon per million mapped fragments.** Fig. S5. **The expression patterns of orthologous AP2/ERF genes between pepper and tomato under cold, heat, and salt stresses. **A** The heatmap shows expression profiles of orthologous genes. The scale bar on the bottom represents FPKM values. **B** Pearson’s correlation analysis between orthologous genes in pepper and tomato.

## Data Availability

All data generated or analysed during this study are included in this published article and its supplementary information files.

## Abbreviations

AP2: APETALA2
ERF: Ethylene-Responsive Factor
ANT: AINTEGUMENTA
DREB: Dehydration-Responsive Element-Binding protein
GO: Gene ontology
DEG: Differentially expressed gene
FPKM: Fragment Per Kilobase of transcript per Million mapped reads
FDR: False discovery rate
