# Supplementary material for: Comparative and expression analyses of AP2/ERF genes reveal copy number expansion and potential functions of ERF genes in Solanaceae
Source: BMC Plant Biol. 2023 Jan 23;23:48. doi: 10.1186/s12870-022-04017-6 (PMC9869560; doi:10.1186/s12870-022-04017-6)
Supplement: Supplementary file 2 — Additional file 2: Fig. S1. Chromosome allocation of pepper, tomato and potato AP2/ERF genes. White rectangular boxes indicate chromosomes. Fig. S2. Syntenic analyses of all pepper, tomato and potato chromosomes. Colored rectangles represent the chromosomes (chr) of each species. The genes with orthologous relationships among the three species are linked with lines. The line color indicates details of the orthologous relationships, as indicated. Fig. S3. Expression profiles of pepper AP2/ERF genes under various abiotic stresses. A Heat map showing the relative differences of expression of AP2/ERF genes under abiotic stress. The heat map values (log2 fold-change) are calculated by FPKM (abiotic stress) /FPKM (control). The names of the AP2/ERF genes are displayed next to the heat map. The normalized values of the heat map are represented in the scale bars, positioned on the right side of the heat map; green implies a low level of expression, whereas red represents a high level of expression. B The distribution of AP2/ERF DEGs in the subgroups is illustrated in the heat map (inset). FPKM, fragments per kilo base of exon per million mapped fragments. Fig. S4. The expression value of tomato AP2/ERF genes under various abiotic stresses. A The heat map represents the value of expression of AP2/ERF genes under abiotic stress. The values are calculated by log2 fold-change of tomato AP2/ERF genes and fold-change values are calculated by dividing FPKM from each stress to control (C: Cold, D: Drought, H: Heat, S: Salt). The colored bar positioned on the right represents the following range of expression levels:-3 (green) to +3 (red). B The number of tomato AP2/ERF DEGs in are displayed in heat map (inset). FPKM, fragments per kilo base of exon per million mapped fragments. Fig. S5. The expression patterns of orthologous AP2/ERF genes between pepper and tomato under cold, heat, and salt stresses. A The heatmap shows expression profiles of orthologous genes. The scale bar on the [file 12870_2022_4017_MOESM2_ESM.pdf]

Supplementary figure 1

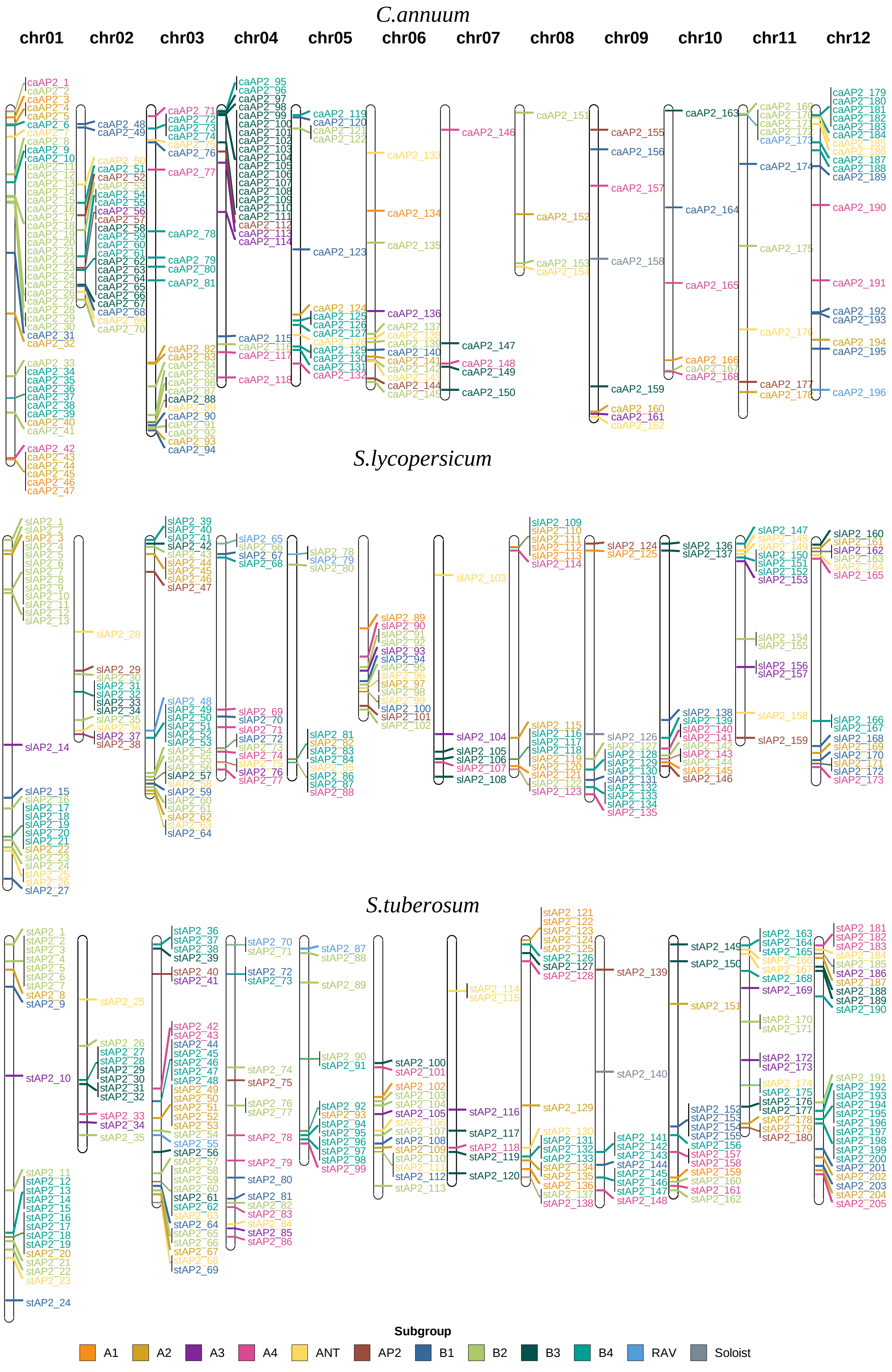

Fig. S1. Chromosomal location of pepper, tomato, and potato AP2/ERF genes. White rectangular boxes indicate chromosomes.

Supplementary figure 2

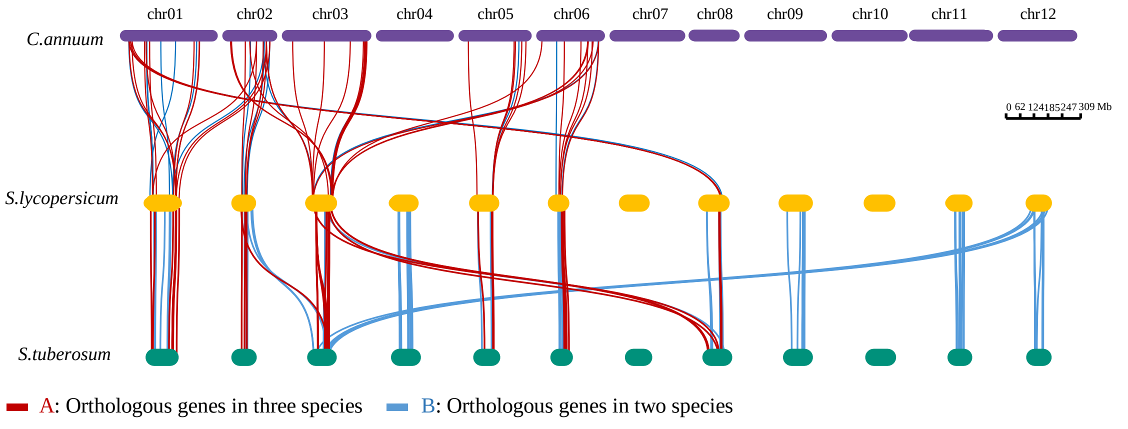

Fig. S2 Syntenic analyses of all pepper, tomato, and potato chromosomes. Colored rectangles represent the chromosomes (chr) of each species. The genes with orthologous relationships among the three species are linked with lines. The line color indicates details of the orthologous relationships, as indicated.

Supplementary figure 3

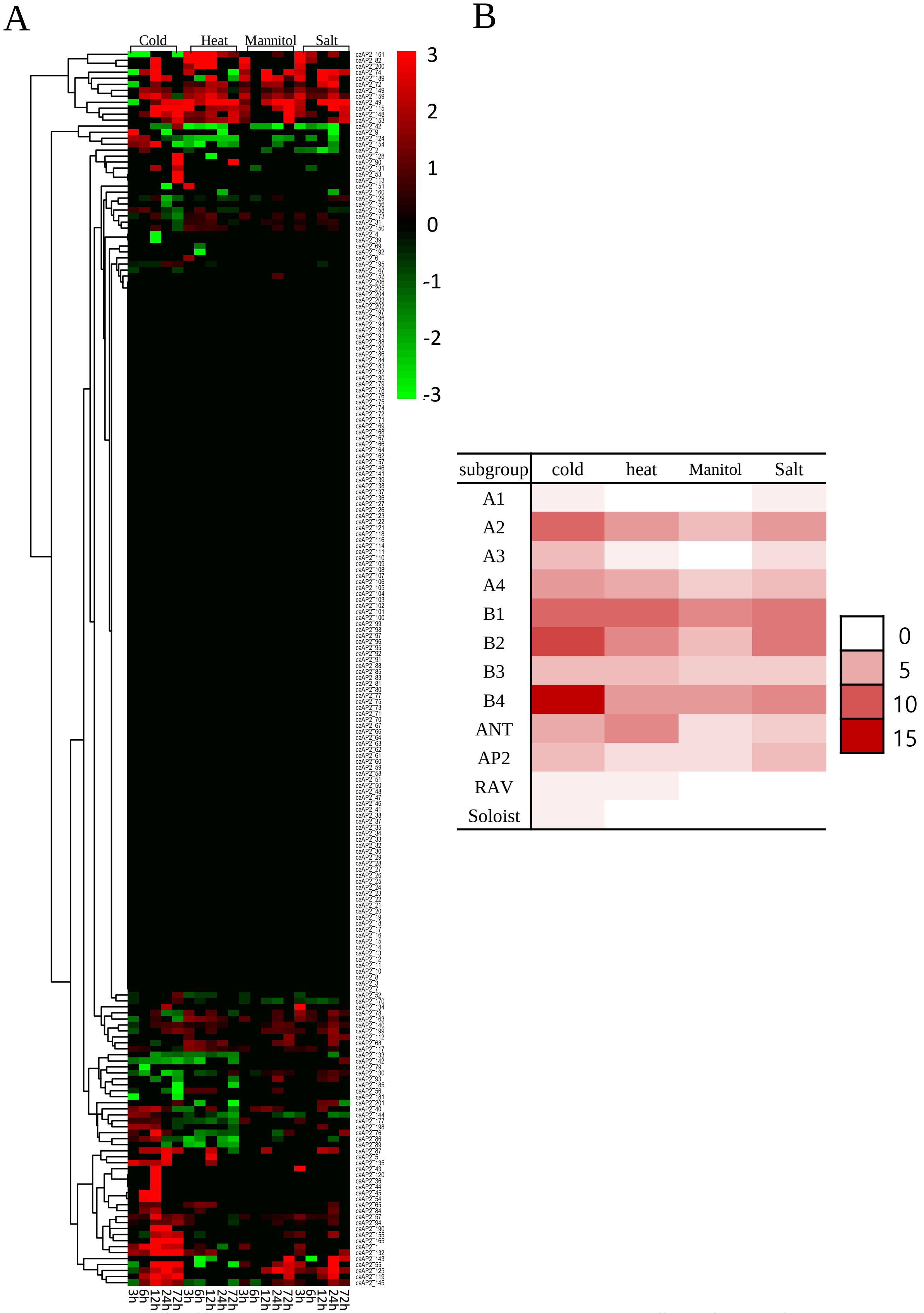

Fig. S3 Expression profiles of pepper AP2/ERF genes under various abiotic stresses. (A) Heat map showing the relative differences of expression of AP2/ERF genes under abiotic stress. The heat map values (log2 fold-change) are calculated by FPKM(abiotic stress)/FPKM(control). The names of the AP2/ERF genes are displayed next to the heatmap. The normalized values of the heat map are represented in the scale bars, positioned on the right side of the heatmap; green implies a low level of expression, whereas red represents a high level of expression. (B) The distribution of AP2/ERF DEGs in the subgroups is illustrated in the heatmap (inset). FPKM, fragments per kilobase of exon per million mapped fragments.

Supplementary figure 4

A

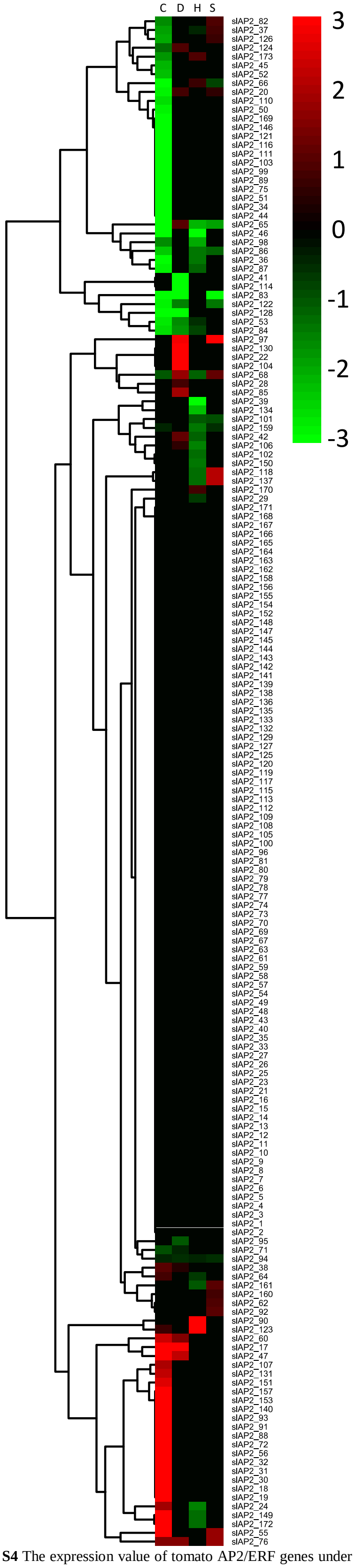

B

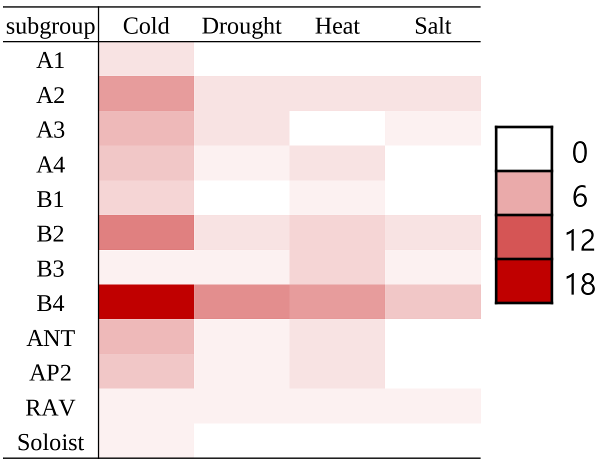

Fig. S4 The expression value of tomato AP2/ERF genes under various abiotic stresses. (A) The heatmap represents the value of expression of AP2/ERF genes under abiotic stress. The values are calculated by log2 fold-change of tomato AP2/ERF genes and fold-change values are calculated by dividing FPKM from each stress to control (C: Cold, D: Drought, H: Heat, S: Salt). The colored bar positioned on the right represents the following range of expression levels: -3 (green) to +3 (red). (B) The number of tomato AP2/ERF DEGs in are displayed in heatmap (inset). FPKM, fragments per kilobase of exon per million mapped fragments.

Supplementary figure 5

A

Subgroup	Pepper ID	Cold	Heat	Salt	Tomato ID	Cold	Heat	Salt
A4	caAP2_1				slAP2_123			
B2	caAP2_2				slAP2_122			
A1	caAP2_3				slAP2_121			
A2	caAP2_4				slAP2_22			
B4	caAP2_6				slAP2_18			
B2	caAP2_8				slAP2_1			
B4	caAP2_9				slAP2_17			
B2	caAP2_15				slAP2_7			
B2	caAP2_17				slAP2_10			
B2	caAP2_26				slAP2_11			
B2	caAP2_27				slAP2_10			
B1	caAP2_31				slAP2_15			
A2	caAP2_32				slAP2_3			
B2	caAP2_33				slAP2_16			
B4	caAP2_34				slAP2_17			
B4	caAP2_38				slAP2_21			
A2	caAP2_40				slAP2_22			
B1	caAP2_48				slAP2_64			
B1	caAP2_49				slAP2_59			
ANT	caAP2_50				slAP2_28			
B4	caAP2_51				slAP2_53			
AP2	caAP2_52				slAP2_29			
B2	caAP2_53				slAP2_1			
B4	caAP2_54				slAP2_31			
AP2	caAP2_57				slAP2_47			
B3	caAP2_58				slAP2_42			
B4	caAP2_60				slAP2_32			
B4	caAP2_61				slAP2_21			
B3	caAP2_63				slAP2_33			
ANT	caAP2_69				slAP2_36			
B4	caAP2_73				slAP2_39			
B4	caAP2_79				slAP2_41			
A2	caAP2_82				slAP2_44			
B2	caAP2_85				slAP2_54			
B2	caAP2_86				slAP2_55			
B2	caAP2_87				slAP2_56			
B3	caAP2_88				slAP2_57			
ANT	caAP2_89				slAP2_58			
B1	caAP2_90				slAP2_59			
B2	caAP2_91				slAP2_60			
B2	caAP2_92				slAP2_61			
A2	caAP2_93				slAP2_62			
B1	caAP2_94				slAP2_64			
B2	caAP2_121				slAP2_80			
A2	caAP2_124				slAP2_82			
B4	caAP2_126				slAP2_83			
ANT	caAP2_128				slAP2_85			
B4	caAP2_129				slAP2_87			
A4	caAP2_132				slAP2_88			
ANT	caAP2_133				slAP2_63			
A1	caAP2_134				slAP2_89			
B2	caAP2_135				slAP2_91			
A3	caAP2_136				slAP2_93			
ANT	caAP2_138				slAP2_58			
B2	caAP2_139				slAP2_56			
A2	caAP2_141				slAP2_97			
B2	caAP2_142				slAP2_55			
AP2	caAP2_144				slAP2_47			
B2	caAP2_145				slAP2_43			

0 50 140   0 50 120

B

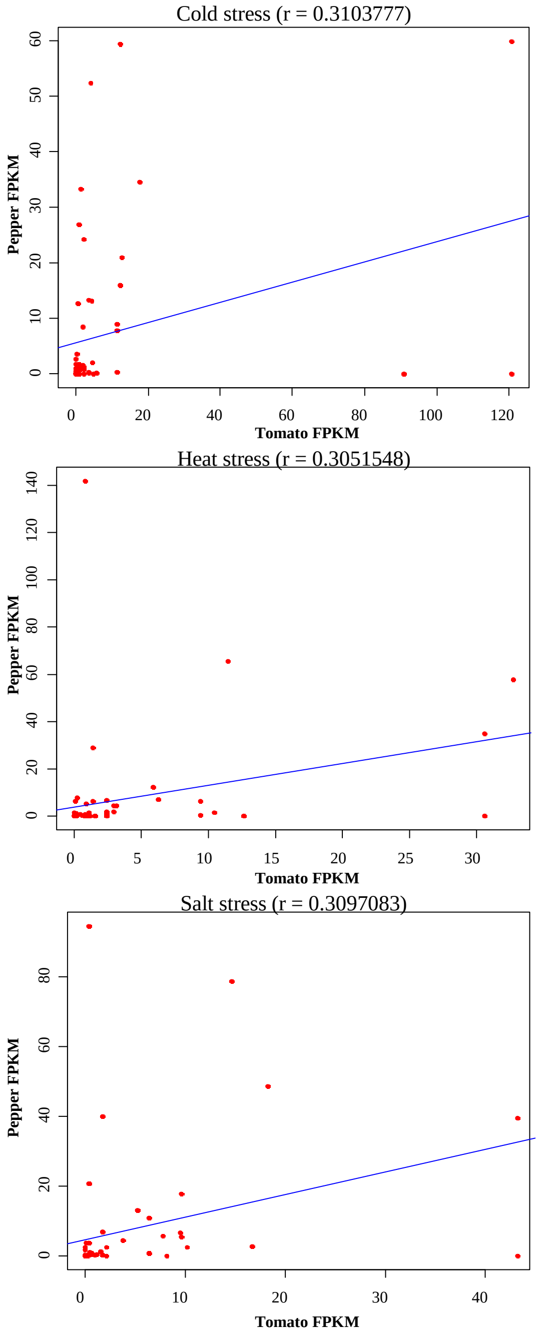

Fig. S5 The expression patterns of orthologous AP2/ERF genes between pepper and tomato under cold, heat, and salt stresses. (A) The heatmap shows expression profiles of orthologous genes. The scale bar on the bottom represents FPKM values. (B) Pearson's correlation analysis between orthologous genes in pepper and tomato.
